# Mediating Role of Moral Disengagement between Emotional Manipulation and Psychological Well-Being: Does Age Matter?

**DOI:** 10.3390/bs11090117

**Published:** 2021-08-31

**Authors:** Syeda Rubab Aftab, Jamil Ahmad Malik

**Affiliations:** National Institute of Psychology, Quaid i Azam University, Islamabad 45401, Pakistan; ja.malik@nip.edu.pk

**Keywords:** emotional manipulation, moral disengagement, psychological well-being, moderated mediation, age

## Abstract

**Background/Aims**: When people hone their emotional skills, they become better at manipulating others. They use their emotional skills for coping with the demands of life. This study investigated the mediating role of moral disengagement between emotional manipulation and psychological well-being. Further, the moderating role of age is tested for the mediation model of the study. **Methods**: This study has a cross-sectional design. Participants included students from private and public institutions (*n* = 542; Mean age = 18.59 years, SD = 2.10 years; gender = 46% males). Responses were collected on emotional manipulation, moral disengagement, and psychological well-being questionnaires. Analyses were conducted using SPSS 21 and PROCESS 3.1. **Results**: The correlation analysis showed that both in late adolescents and young adults, moral disengagement negatively correlated with psychological well-being. However, the correlation is much stronger for young adults as compared to late adolescents. Similarly, emotional manipulation has a stronger positive correlation with moral disengagement in young adults compared to late adolescents. Results also showed that moral disengagement and emotional manipulation is higher in males than females, and psychological well-being is higher in females than males. Moral disengagement appeared to be a negative mediator for the relationship between emotional manipulation and psychological well-being. Further, age moderated the indirect effect of emotional manipulation on psychological well-being through moral disengagement. The moderation of age suggests that young adults are more inclined toward moral disengagement behaviors for manipulating emotions in comparison to late adolescents. **Conclusions**: It is concluded that use of emotional manipulation is associated with a direct increase in psychological well-being; however, indirect emotional manipulation decreases psychological well-being, with an increased use of moral disengagement. Moreover, this indirect effect is stronger in young adults compared to late adolescents, as young adults are more inclined toward moral disengagement.

## 1. Introduction

According to theories of emotions, people who manage their emotions well are more able to cope with the challenges of life. This quality also helps to determine the stressful situation and to bounce back from setbacks. These people usually feel good about themselves and are able to maintain good relationships. However, the need to advance on personal gains motivates individuals to emotionally manipulate others for their self-serving biases, known as the intrinsic component of mental health [1,2].

Researchers in the field of positive psychology recognize that optimal human functioning requires more than the absence of risk or pathology, and have been seeking to identify factors that contribute to the development of well-being [3,4,5,6]. The intelligent use of emotions is one of the fundamental mechanisms [7] that enhance coping resources to promote well-being [8,9,10,11]. An individual who is capable of managing emotions has sufficient self-awareness to recognize negative feelings, and thus reasons accordingly to prevent intensification. This is a key ability in maintaining good well-being. The lack of this ability leads to uncontrolled and misunderstood emotions, which can easily intensify one’s vulnerability to negative consequences [12].

According to Goleman [13], to learn to behave in an optimal way (no matter how a particular situation is), one needs to remember how one’s emotions are formed. Therefore, emotional intelligence is a matter of concern for individuals’ well-being.

Emotional intelligence is the capability to monitor one’s own and another’s emotions to guide thinking and behavior, and to manage and adjust emotions in order to adapt to the environment or to achieve one’s goals [14,15]. Recent studies have identified a variety of contexts where emotional intelligence is not helpful, and may have negative interpersonal and intra personal consequences. For instance, individuals can make use of their high-level capabilities to read and manage others’ emotions to manipulate others’ behavior to suit their own interests. Austin and his colleagues [16] extended this notion of emotional intelligence, arguing that the ability to use and manage emotions could also be used in negative and malicious contexts for self-serving purposes. This misuse of emotional manipulation promotes the objectives of the regulator rather than those of the target [17,18], which is the dark side of emotional intelligence. Hence, a component of emotional intelligence is the emotional manipulation that is the capability of managing others’ emotions for self-interest or the use of positive emotional skills for darker purposes [16,19,20].

In their review pertaining to emotional intelligence, health, and well-being, Zeidner and colleagues [21] suggest that emotional intelligence influences well-being by fostering adaptive methods of coping with social challenges, social stress, and interpersonal conflicts; promoting the development of supportive social networks; decreasing negative and increasing positive emotions; and enhancing emotional regulation. Emotional intelligence is also conceptually related to psychological well-being as it focus on personal growth and self-actualization [21]. Low emotional intelligence is related to vulnerability to other mental health issues, such as stress, anxiety, and depression [12,22]. Empirical evidence has also shown that emotional intelligence serves as a coping strategy to enhance subjective well-being [23,24,25,26,27,28] and plays a significant role in mental health [9,29]. Emotional intelligence has gained much attention as a focus of research and intervention to develop a set of skills that can be taught to enhance coping resources and to promote well-being [8,9,10,11,30]. However, the use of emotional intelligence in manipulative and antisocial ways is a neglected area of research [16,31]. Emotional manipulators lack moral regard [32,33], and their self-serving tendencies may make them likely to behave maliciously. However, if they see their actions as unethical, it negatively affects their well-being [34,35]. In addition, principles and values may also influence an individual’s use of emotional manipulation [20]. Therefore, it is not clear whether someone with the ability to manipulate will actually choose to engage in manipulative tactics. To answer this, moral disengagement is a relevant cognitive factor that may help to better understand the immoral behaviors of individuals [36].

Moral disengagement is the cognitive process responsible for individuals’ justification regarding their immoral actions and behavior [37]. According to Bandura, individuals’ moral standards guide their conduct, and accordingly, they tend to engage in behavior that brings them self-worth and satisfaction while refraining from behavior that violates those standards and results in degradation, guilt, and shame. However, Bandura [38] also highlighted that these internal standards are not constantly active, and people can temporarily silence their moral standards in order to engage in conduct that they would generally consider reprehensible, reducing cognitive dissonance between their internal moral system and their actual behavior. The process of moral disengagement is a multi-functional regulatory and coping mechanism that not only allows individuals to engage in unethical behavior, but also manage the emotions that may arise from learning the consequences of such behavior. Researchers have focused on emotional reactions as possible indicators of moral disengagement [39,40]. Moreover, individuals decrease their own well-being when their actions violate the basic beliefs they hold about the world and of themselves (moral standards) [41]. This discrepancy in their ideal and actual behaviors increases distress and is self-punitive [42,43]. The temptation to emotionally manipulate persuades individuals to seek, rationalize, and validate their behavior by disengaging self-sanctions from their anti-social behavior, allowing them to maintain a sense of well-being, which has not yet been investigated. Therefore, in the present study, we hypothesized that the mediating role of moral disengagement exacerbates the negative consequences of emotional manipulation on psychological well-being.

The literature shows evidence of significant differences across various age groups regarding management and understanding of emotions, moral disengagement, and psychological well-being. According to Grieve and Panebianco [44], the predictive pattern of manipulative behavior varies across sex and age. The ability to manage and understand emotions increases with age [13,45,46,47]. Kaufman [48] explained this phenomenon as being a result of lifelong learning and accumulated knowledge. This ability improves with practice [49] as people gain experience with emotional intelligence. The ability to understand emotions increases with age; therefore, younger adults may be better in emotion regulation strategies than younger ones [50,51,52]. Associations between age and moral disengagement have also been addressed in various research studies. The literature shows that moral disengagement decreases with age [53]. The same findings are supported by others who report that moral judgment increases from late adolescence into early adulthood [54]. Given such a critical role of age, in the present study, we hypothesized that age moderates the mediational link between emotional manipulation, moral disengagement, and psychological well-being. More specifically, we hypothesized a stronger indirect effect of emotional manipulation on psychological well-being through moral disengagement for younger adults as compared to late adolescents.

## 2. Method

### 2.1. Participants and Procedure

Participants with ages ranging from 16 to 25 years (*n* = 542; Mean age = 18.59 years, SD = 2.10 years; gender = 46% males) were included from various public and private colleges and universities after formal permission from authorities and informed consent form participants and their parents/legal representatives (where applicable). Participants were divided into two age groups to form an adolescent group of *n* = 355 participants aged 16–18 (M = 17.33, SD = 0.54, 53.8% women) and a young adult group of *n* = 189 participants aged 19–25 years (M = 20.97, SD = 1.89, 52.4% women). The participants were approached and the purpose of the study was explained while reassuring them that their participation was voluntary, and that the data collected will be treated as confidential. After giving written informed consent, participants completed questionnaires. To ensure the quality and reliability of data, the returning 560 questionnaires were screened for outliers, and 18 cases where participants did not fill the booklet in properly were discarded. After screening the data, analyses were conducted using SPSS version 21. To see the relationship patterns among the study variables, the Pearson bivariate correlation, independent sample *t*-test, Mediation and conditional process analysis using Process macro were conducted separately for both late adolescents and young adults.

### 2.2. Instruments

#### 2.2.1. Emotional Manipulation Scale

The adapted version of the Emotional Manipulation Scale [16] was used. It includes eight items with a 5-point Likert type scale (1 = strongly disagree; 5 = strongly agree). Sample items include “I know how to embarrass someone to stop them behaving in a particular way”. Higher scores indicate a higher tendency of emotional manipulation. In the present study, the alpha reliability for the scale is α 0.80. Previous studies showed a high alpha reliability of the emotional manipulation scale with Cronbach’s α = 0.87 to 0.90 [16,20,55].

#### 2.2.2. Moral Disengagement Scale

The Moral Disengagement Self-Report Questionnaire [56] includes 32 items, with a 5-point Likert type scale (1 = strongly disagree, to 5 = strongly agree). An example is: “Stealing some money is not too serious compared to those who steal a lot of money”. The scale consists of eight dimensions and the composite scale score represents overall moral disengagement. For the present study, the composite score was used. A higher score indicates a higher level of moral disengagement. In the present study, the alpha reliability for the scale is α 0.81. Previous studies showed high alpha reliability for a composite measure of moral disengagement, that is, α = 0.86 [56,57,58].

#### 2.2.3. Psychological Well-Being Scale

Ryff’s [58] Psychological Well-Being Scale consists of 42 items with a six-point rating scale (1 = strongly disagree, to 6 = strongly agree). A sample item is: “I think it is important to have new experiences that challenge how you think about yourself and the world”. The scale consists of six dimensions, and the composite scale score represents overall psychological well-being. For the present study, the composite score was used. A higher score indicates a higher level of psychological well-being. In the present study, the alpha reliability for the scale is α 0.80. Previous studies showed good internal consistency with alpha coefficients, with scores ranging from 0.81 to 0.88 [59,60,61].

## 3. Results

The correlation analysis (Table 1) showed that for both late adolescents and young adults, emotional manipulation was positively associated with moral disengagement, and moral disengagement was negatively associated with psychological well-being. However, these associations were higher for young adults in comparison to late adolescents. Further, the descriptive statistics showed that moral disengagement was high and psychological well-being was low among young adults as compared to late adolescents. However, in emotional manipulation, there was no significant difference.

Regarding gender differences, the correlation matrix showed that emotional manipulation is negatively and significantly related to gender (higher in males) only in late adolescents; however, in young adults, the difference was not significant across gender. Moral disengagement is negatively (higher in males), and psychological well-being is positively (higher in females) related to gender among both late adolescents and young adults. These results were further confirmed by an independent sample *t*-test (Table 2), suggesting that females are low on emotional manipulation and moral disengagement, and scored better psychological well-being as compared to their male counterparts. The effect size, as indexed by Cohen’s *d*, was small 0.23 for emotional manipulation, and small to medium (0.35 and 0.32, respectively) for emotional manipulation and moral disengagement.

**Figure 1:**
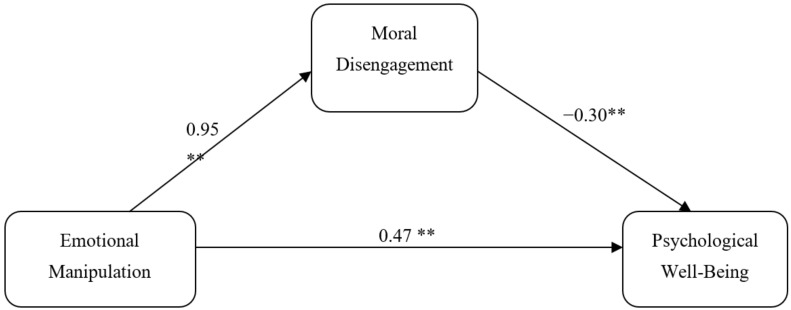
Mediating effect of moral disengagement for the relationship between emotional manipulation and psychological well-being, ** *p* < 0.01.

Mediation analysis was conducted using Process macro (Model 4) in order to estimate the indirect effect of moral disengagement on the relationship between emotional manipulation and psychological well-being (Figure 1). The results (Table 3) demonstrated that emotional manipulation increased psychological well-being (B = 0.47, *p* > 0.000); however, it also increased moral disengagement (B = 0.95, *p* > 0.000), which in turn decreased psychological well-being (B = −0.30, *p* < 0.01). This resulted in a significantly negative indirect effect of emotional manipulation on psychological well-being (B *Indirect* = −0.24, CL: −0.41 to −0.19).

**Figure 2:**
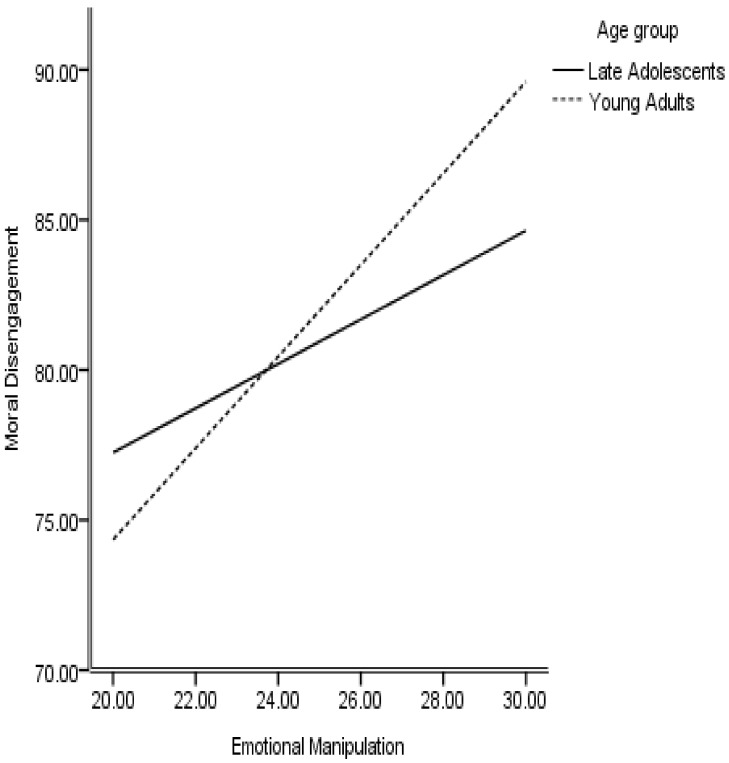
Age moderating between Emotional Manipulation and Moral disengagement.

**Figure 3:**
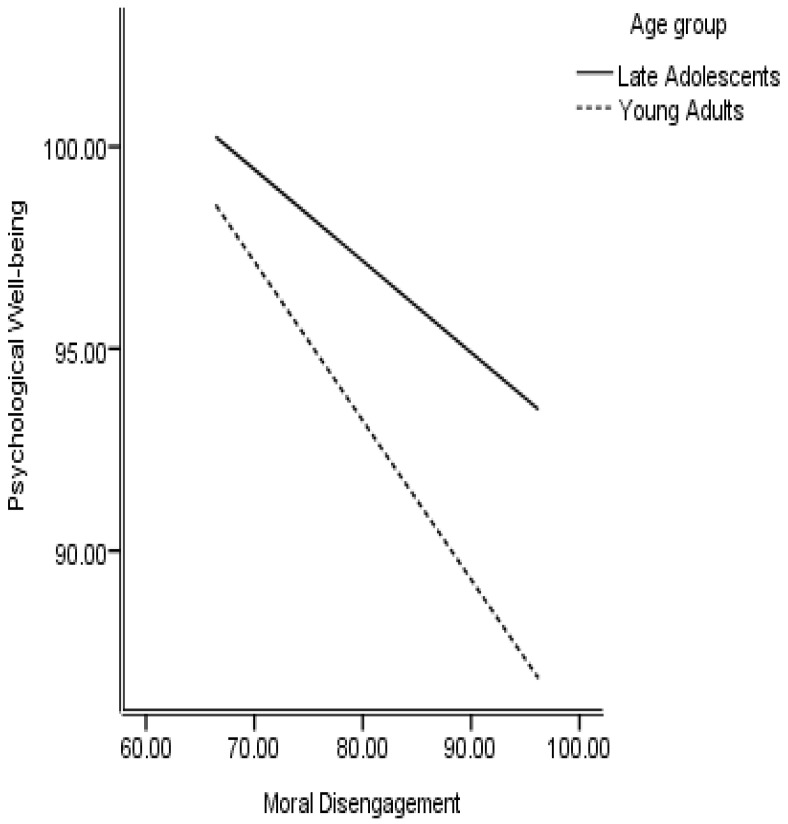
Age moderating between Moral disengagement and Psychological well-being.

The conditional process analysis was conducted through Process macro (Model 59) to test the conditional indirect effect of emotional manipulation on psychological well-being through moral disengagement, with age as a moderator. The conceptualized moderated mediation model appeared to be a good fit to the data with a significant index of moderated mediation = −0.43, 95% CI (−0.75, −0.19), suggesting that the mediating role of moral disengagement is different for late adolescents as compared to young adults. The effect of emotional manipulation on moral disengagement is moderated by age with a positive interaction (B = 0.79, *p* < 0.000, Figure 2). Further, the effect of moral disengagement on psychological well-being is also moderated by age (B interaction = −0.17, *p* = 0.05, Figure 3). However, the interaction of the direct effect of emotional manipulation on psychological well-being was not statistically significant (B = 0.12, *p* = 0.69) suggesting that emotional manipulation skills are effective in maintaining psychological well-being across both age groups. The results presented in Table 4 suggest that negative consequences of moral disengagement cost more to young adults in terms of psychological well-being as compared to older adolescents.

## 4. Discussion

The present study aimed to examine the mediating role of moral disengagement for the relationship between emotional manipulation and psychological well-being. Further, the mediation model was tested for the moderating role of age. The purpose of the study was to provide a quantitative assessment for the relationship between emotional manipulation and psychological well-being mediating through moral disengagement and to test the moderating role of age. Though preliminary analysis showed a positive relationship between emotional manipulation and psychological well-being, contrary to the study’s assumption, bivariate correlation across age groups suggested a non-significant relationship between emotional manipulation and psychological well-being in both age groups (i.e., older adolescents and young adults). These results are also contradictory to the existing literature that has shown negative associations between unethical behavior and well-being [62,63,64,65,66]. This may be due to the fact the simple bivariate correlations do not validate complicated social and behavioral patterns involving multiple factors. As was expected, the results supported a positive association between emotional manipulation and moral disengagement. These results are in line with the prior findings that argued that emotional manipulators have no moral regard [32,33]. The results suggested people with the ability to engage in emotional manipulation use moral disengagement to legitimize and justify their behavior.

We found adolescent males to be higher than adolescent females on emotional manipulation; however, these differences disappeared among young adults, suggesting that gender roles are not stable in determining emotional manipulation behavior across different ages. However, males appeared to be significantly higher on moral disengagement and lower on psychological well-being across both age groups. The empirical literature has consistently reported that males score higher on moral disengagement than females [55,67,68,69,70,71].

The mediation analysis showed that moral disengagement negatively mediated the effects of emotional manipulation on psychological well-being. The psychological well-being of emotional manipulators ultimately deteriorates as they legitimize and justify their manipulative behavior without self-condemnation. This also validates our explanation regarding the unreliable representation of the correlation analysis for testing a complicated behavioral pattern involving multiple factors. The present study results are supported by the earlier findings reporting that people judge themselves in moral terms and unethical actions are (in general) that are antithetical to well-being and happiness [62]. Unethical behaviors are likely to have negative consequences for psychological well-being [64,72] that may be induced by the individual’s own actions on the basis of biased personnel decisions [73]. According to Byrne [41], individuals’ own well-being decreases when their actions violate the basic beliefs they hold of the world and of themselves (moral standards). This discrepancy between their ideal and actual behaviors [42,74] may cause distressed and self-punitive feelings.

Further, the moderated mediation showed that the conditional direct effect of emotional manipulation on psychological well-being was significantly positive for both late adolescents and young adults. This suggests that age does not matter in using emotional manipulative skills for maintaining psychological well-being. However, the pattern showed that with increasing age, emotional manipulators are better able to use their emotional manipulation skills. The present findings are consistent with previous evidence which showed that emotional management skills of others and themselves are improved with practice [48]. The simple explanation means that older people can be more skilled, as they have more opportunities to practice their skills. Therefore, older adults have a better understanding of emotion regulation strategies than younger adults [49,50,75].

Our results from the conditional indirect effect are in line with the social cognitive theory [37,38], which states that changes in moral disengagement and immoral behavior is a gradual process over time. The results also revealed that age plays a critical role when moral disengagement is taken into account in assessing the relationship between emotional manipulation and psychological well-being. Age significantly moderated the indirect effect of emotional manipulation on psychological well-being by means of moral disengagement. The conditional indirect effect is negative for both late adolescents and young adults; however, the strength of this negative indirect effect increases with age. This negative indirect effect is exponentially increased for younger adults in comparison to older adolescents. The moderated mediation model showed that in summary, the effect of emotional manipulation through moral disengagement decreases psychological well-being. Compared to late adolescents, young adults reported lower levels of psychological well-being when they are high in emotional manipulation, and justified their manipulative behavior by deactivating their self-regulated mechanisms and moral self-sanctions. Evans and colleagues [76] concluded the same with the arguments that those individuals who behave unethically are not necessarily comfortable with their actions. The present study results further confirm this argument.

## 5. Conclusions

The present study elaborated the role of moral disengagement in the relationship between emotional manipulation and psychological well-being. The findings are helpful in clarifying discrepancies for the effect of emotional manipulation on psychological well-being across adolescents and young adults. The findings are in line with the social cognitive theory and elaborate the developmental nature of the study variables. It is evidenced that the apparently positive effect of emotional manipulation on psychological well-being actually has negative underlying consequences when the mediating role of moral disengagement is investigated. Further, young adults are at higher risk of facing underlying negative consequences. The findings are useful in the development of intervention strategies, particularly for the at-risk age group, that is, young adults. It is suggested that emotional manipulation shall be carefully observed, particularly among young adults, and intervention plans shall be devised using strategies to counter moral disengagement to avoid negative consequences on the psychological well-being of the at-risk population.

Though the current study adds to the literature, it has limitations as well. First, a convenience sample was utilized, which can potentially limit the generalizability of the results. Moreover, the study attempted to address this concern by utilizing a correlational study. Yet, future work should consider a longitudinal design to see the long-term effects of moral disengagement and emotional manipulation on psychological well-being with greater demographic diversity. The present study’s results should be evaluated with cultural aspects and other individual and situational factors influencing the relationship, such as social relationships and family independence, that lead to changes in psychological well-being. The findings of this study may contribute to the literature and works on cultural, behavioral, social, positive, and health psychology.

## Figures and Tables

**Table 1:** Descriptive statistics and Correlation Coefficient of Study Variables (*n* = 542).

S. no		M	SD	1	2	3	4	5
1	Gender			-	0.02	−0.17 **	−0.15 **	0.17 **
2	Age	17.33	0.54	−0.16 *	-	−0.06	0.03	−0.18 **
3	Emotional Manipulation	25.24	5.52	−0.07	0.12	-	0.28 **	0.10
4	Moral Disengagement	80.95	14.12	−0.18 *	0.20 **	0.41 **	-	−0.16 **
5	Psychological well-being	96.89	15.52	0.17 *	−0.21 **	−0.03	−0.42 **	-
	M				20.97	25.22	82.26	92.43
	SD				1.9	4.66	16.79	13.43

* *p* < 0.05, ** *p* < 0.01. Note: Upper diagonal: Late adolescents; Lower diagonal: Young adults; Male = 0; Female = 1.

**Table 2:** Differences on study variables across Gender (*n* = 542).

	Gender								
	Male (*n* = 250)		Female (*n* = 290)				95% CI		
Variables	M	SD	M	SD	t	*p*	LL	UL	Cohen’s *d*
EM	25.86	5.09	24.68	5.16	3.19	0.000	0.55	2.31	0.23
MD	84.15	14.08	79.34	15.63	3.72	0.000	2.27	7.35	0.32
PWB	92.39	14.39	97.68	15.15	4.07	0.000	−7.71	−2.69	0.35

Note: EM = Emotional manipulation, MD = Moral disengagement, PWB = Psychological well-being.

**Table 3:** Mediating effect of moral disengagement for the relationship between emotional manipulation and psychological well-being (*n* = 542).

			95% CL	
Conditions	B	*p*	LL	UL
EM------> MD	0.95	0.000	0.71	1.18
EM------> PWB	0.47	0.000	0.22	0.72
MD ------> PWB	−0.30	0.000	−0.39	−0.22
EM ----->MD----->PWB	−0.24		−0.41	−0.19
R^2^	0.09			
F	25.35	0.000		

Note: EM = Emotional manipulation, MD = Moral disengagement, PWB = Psychological well-being.

**Table 4:** Conditional Direct and Indirect effects of Emotional Manipulation on Psychological Well-being through Moral Disengagement, moderated by Age (*n* = 542).

Predictors	Moderator Levels	Mediator		Psychological Well-Being		
		MD		B	95% CI	
		B	*p*		LL	UL
Emotional manipulation		1.01	0.000	0.49	0.23	0.74
Moral Disengagement				−0.28	−0.37	−0.20
Age		1.22	0.34	−4.21	−6.74	−1.67
Emotional manipulation*Age		0.79	0.003	0.12	−0.46	0.69
	Late Adolescents			0.45	0.16	0.73
	Young Adults			0.56	0.06	1.06
Moral Disengagement*Age				−0.17	−0.34	0.01
	Late Adolescents			−0.17	−0.29	−0.08
	Young Adults			−0.60	−0.91	−0.38

## Data Availability

The data that support the findings of this study are available upon request from the corresponding author.
